# OR10-006 - Canakinumab in patients with TRAPS

**DOI:** 10.1186/1546-0096-11-S1-A189

**Published:** 2013-11-08

**Authors:** HJ Lachmann, L Obici, A Meini, V Tormey, K Abrams, N Davis, C Andrews, SG Bhansali, M Gattorno

**Affiliations:** 1 University College London Medical School, London, UK; 2 IRCCS Fondazione Policlinico San Matteo, Pavia, Italy; 3 Pediatric Immunology and Rheumatology, Brescia, Italy; 4 Galway University Hospitals, Galway, Ireland; 5 Novartis Pharmaceuticals Corporation, East Hanover, NJ, USA; 6 Novartis Pharmaceuticals UK Limited, Surrey, UK; 7 G Gaslini Institute, Genova, Italy

## Introduction

TNF-receptor associated periodic syndrome (TRAPS) is a rare, dominantly inherited periodic fever syndrome due to mutations of the *TNFRSF1A* gene. The IL-1 receptor antagonist anakinra has been reported to be an efficacious daily treatment. Canakinumab (CAN) is a fully human monoclonal selective anti-IL-1β antibody with a T_1/2_ of ~4 wks. Interim clinical and PK data of CAN treatment in patients with active TRAPS are presented.

## Objectives

To assess the efficacy, PK, and safety of canakinumab in patients with active TRAPS.

## Methods

14 adults and 6 children (7-78 yrs) with active TRAPS entered a 3-part trial: 4 months open-label 150 mg (or 300 mg) CAN every 4 wks followed by up to 5 months treatment withdrawal, then 24 months open-label CAN. Primary endpoint was complete or almost complete response at Day 15 based on physician assessed absent or minimal TRAPS signs/symptoms and normal or ≥70% reduced CRP and/or SAA. Those without response by Day 8 were eligible for another 150 mg dose and then 300 mg thereafter. Patients were observed after last dose until relapse (5 month max) before restarting CAN. Population PK analysis was performed using NONMEM based on CAN concentrations determined by ELISA from blood samples collected at pre-specified times points during the first month, at each pre-dose of CAN, and at flares thereafter.

## Results

At Day 15, 19 (95%) patients achieved complete/almost complete response, including all 4 patients without it at Day 8. Two patients were dose up titrated. Clinical remission was maintained by all from Day 15 onwards except 1 who relapsed at Day 85 (during 4 month treatment period), responding to that visit’s CAN dose. Upon CAN withdrawal, all patients relapsed after a median of 92 days (range 72-122 days). 18 regained response 8-27 days after restarting CAN and 2 relapsed at final visit following last dose administered during treatment period without follow-up at time of this analysis. Population PK analysis showed that serum clearance and volume of distribution of CAN were dependent on bodyweight. The estimated apparent serum clearance (CL/F) was 0.238±0.0139 L/day and the corresponding volume of distribution (V_ss_/F) was 8.06 L. Following the first dose, mean±SD observed C_max_ was 16.4±4.62 μg/mL and the median T_max_ was 7.4 days. Apparent weight normalized PK parameters were comparable to the PK observed in other indications. All patients reported at least one adverse event (AE); infections, mostly of the upper respiratory tract, (n=15, 75%), followed by headache (n=9) and abdominal pain (n=7). Two serious AEs, an upper respiratory tract infection and a TRAPS relapse, were reported. All patients are ongoing in the trial.

## Conclusion

Canakinumab produced a rapid clinical and serological benefit which was maintained with continued monthly dosing. Relapse occurred at a median of 92 days after last dose and remission achieved upon re-dosing. Weight normalized PK parameters were comparable to PK observed in other indications. Further studies are needed to better define CAN therapy in TRAPS.

## Competing interests

H. Lachmann Consultant for: Novartis, L. Obici Consultant for: Novartis, A. Meini Consultant for: Novartis, V. Tormey: None declared, K. Abrams Shareholder of: Novartis, Employee of: Novartis, N. Davis Employee of: Novartis, C. Andrews Shareholder of: Novartis, Employee of: Novartis, S. Bhansali Shareholder of: Novartis, Employee of: Novartis, M. Gattorno Grant / Research Support from: Novartis, Consultant for: Novartis, Speaker Bureau of: SoBI

